# The positive dimension of schizotypy is associated with a reduced attenuation and precision of self-generated touch

**DOI:** 10.1038/s41537-022-00264-6

**Published:** 2022-06-29

**Authors:** Evridiki Asimakidou, Xavier Job, Konstantina Kilteni

**Affiliations:** Department of Neuroscience, Karolinska Institutet, Solnavägen 9, 17165 Stockholm, Sweden

**Keywords:** Human behaviour, Schizophrenia

## Abstract

The brain predicts the sensory consequences of our movements and uses these predictions to attenuate the perception of self-generated sensations. Accordingly, self-generated touch feels weaker than an externally generated touch of identical intensity. In schizophrenia, this somatosensory attenuation is substantially reduced, suggesting that patients with positive symptoms fail to accurately predict and process self-generated touch. If an impaired prediction underlies the positive symptoms of schizophrenia, then a similar impairment should exist in healthy nonclinical individuals with high positive schizotypal traits. One hundred healthy participants (53 female), assessed for schizotypal traits, underwent a well-established psychophysics force discrimination task to quantify how they perceived self-generated and externally generated touch. The perceived intensity of tactile stimuli delivered to their left index finger (magnitude) and the ability to discriminate the stimuli (precision) was measured. We observed that higher positive schizotypal traits were associated with reduced somatosensory attenuation and poorer somatosensory precision of self-generated touch, both when treating schizotypy as a continuous or categorical variable. These effects were specific to positive schizotypy and were not observed for the negative or disorganized dimensions of schizotypy. The results suggest that positive schizotypal traits are associated with a reduced ability to predict and process self-generated touch. Given that the positive dimension of schizotypy represents the analogue of positive psychotic symptoms of schizophrenia, deficits in processing self-generated tactile information could indicate increased liability to schizophrenia.

## Introduction

Distinguishing between the two causes of our sensory input—the self and the environment—is fundamental for survival. First, it enables the nervous system to detect physically harmful situations for the organism and to act accordingly^1–3^: for example, the touch of a spider crawling up one’s arm (externally generated touch) elicits a dramatically different response from the same touch applied by one’s other hand (self-generated touch). Second, this distinction is a prerequisite for maintaining our self-consciousness and consequently our mental health because it allows us to delimit our own intentions, sensations, actions, thoughts, and emotions from those of others^4–6^. For example, we do not mistake our thoughts for the voices of other people we simultaneously have conversation with, because we attribute the cause of our thoughts to ourselves (self-generated ‘voices’) and the cause of the voices we hear to others (externally generated voices).

How do we make this distinction? The brain uses internal forward models to predict the sensory consequences of movements (corollary discharge) using copies of the motor commands (efference copy)^3,7,8^. These predictions are essential for the fast, online control of movements because they allow the brain to estimate and correct the body’s state despite the inherent delays in the sensory system^3,9–11^. Importantly, these predictions allow the brain to differentiate between self-generated and externally generated sensations: accordingly, those sensations that match the sensory predictions are self-generated, while those that deviate from the predicted ones, or have not been predicted, are attributed to external causes^12^. Moreover, the brain uses these predictions to attenuate the intensity of the self-generated signals, thereby amplifying the difference between self-generated and externally generated information^8,13–15^. In the tactile domain, this attenuation manifests as perceiving self-generated touch as weaker than an externally generated touch of the same intensity^15–26^ and in yielding weaker activity in the secondary somatosensory cortex and the cerebellum^23,27^ and increased functional connectivity between the two areas^23^. This somatosensory attenuation is considered one of the reasons why we cannot tickle ourselves^8,28,29^.

In contrast to healthy individuals, patients with schizophrenia show significantly less attenuation of self-generated tactile sensations at the behavioral level^30^ and do not exhibit attenuation of somatosensory cortical activation for self-generated forces as healthy controls do^31^. Moreover, patients with positive symptoms, such as auditory hallucinations and delusions of control, often fail to attenuate self-generated touch and perceive it as if it were externally generated^32^. Critically, this failure of attenuation is positively correlated with the severity of their hallucinations: the more severe the hallucinations, the lower the somatosensory attenuation^31^.

These findings have supported the neuropsychiatric view that the positive symptoms of schizophrenia can be explained by a deficit in predicting and processing self-generated sensations^33,34^. Such a deficit should hinder the distinction between self-generated and externally generated sensations^35^, reduce the sense of agency^6,36^, and produce perceptual aberrations^37^, including delusions of control^5^ and auditory hallucinations^36^. Consequently, schizophrenia is tightly linked to an atypical perception of self-generated sensations but not externally generated sensations. Despite the heterogeneity of symptoms, schizophrenia has been primarily described as a disorder of the sense of self^38–40^, and self-disorders have been shown to constitute a crucial, trait-like phenotype of the schizophrenia spectrum^41^.

If the positive symptoms of schizophrenia are intrinsically linked to deficits in predicting and processing self-generated somatosensation, then a similar relationship should exist between positive schizotypy and impaired prediction and processing of self-generated somatosensation in nonclinical individuals. Importantly, this approach circumvents many of the methodological confounds arising from patient studies, such as antipsychotic treatment, hospitalization, and disease chronicity, that the patient groups are typically subjected to^42^. Schizotypy, or psychosis-proneness, describes subclinical psychosis-like symptoms or personality characteristics, including peculiar beliefs, unusual sensory experiences and odd behavior^43,44^, that apply to the general population^45–50^. Schizotypal traits are presumed to originate from the same combination of genetic, neurodevelopmental and psychosocial factors as schizophrenia^51–59^, they lie on a continuum with schizophrenia^50^ and are considered a valid phenotypic indicator for the liability to psychosis spectrum disorders and for understanding the underlying psychopathology^45,46,48,57^. Similar to schizophrenia symptom clusters, schizotypy consists of three dimensions, positive, negative, and disorganized^45,50,60^, that broadly correspond to the positive (e.g., hallucinations and delusions), negative (e.g., alogia and apathy) and disorganized symptoms of schizophrenia (e.g., thought disorder and bizarre behavior)^46,61–66^.

Here, we investigated the relationship between schizotypal traits and the perception of self-generated and externally generated somatosensation in 100 healthy individuals. We hypothesized that high positive schizotypy would be associated with reduced somatosensory attenuation and lower precision of self-generated touch.

## Materials and methods

### Participants

The data of one hundred and two participants were used in the present study. Current or history of psychological or neurological conditions, as well as the use of any psychoactive drugs or medication, were criteria for exclusion. All participants reported being completely healthy without neurological or psychiatric disorders or taking any medication to treat such conditions. Our sample size was based on two previous studies assessing the relationship between schizotypy and tactile perception in non-clinical samples^67,68^. The data were pooled from three studies (40, 30, and 32 subjects), all including the same psychophysics task and schizotypy measure, and identical experimental conditions. Two participants were excluded because of missing data in the schizotypy measure. Thus, the final sample consisted of one hundred (100) adults (53 women and 47 men; 91 right-handed, 5 ambidextrous, and 4 left-handed; age range: 18–40 years). Handedness was assessed using the Edinburgh Handedness Inventory^69^. All participants provided written informed consent, and the Swedish Ethical Review Authority (https://etikprovningsmyndigheten.se/) approved all three studies (#2020-03647, #2020-03186, #2020-05457).

### Psychophysical task

**Fig. 1:**
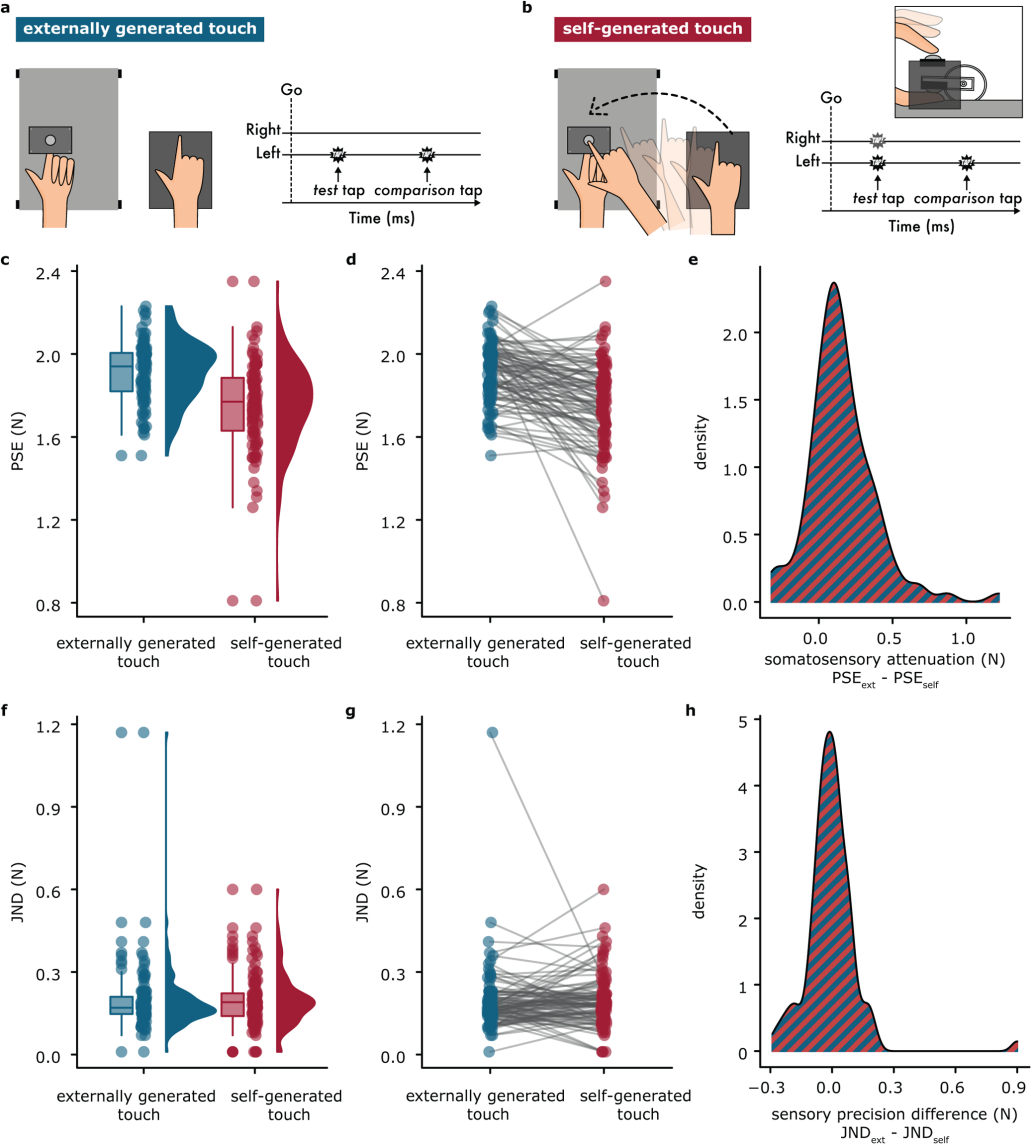
Experimental methods and results. **a, b** The two experimental conditions. **c** The boxplots show the median and interquartile ranges for the PSEs, the jittered points denote the raw data, and the violin plots display the full distribution of the data in each condition. A lower PSE value indicates a lower perceived magnitude. **d** Line plots illustrate the decreases in PSEs when experiencing self-generated tactile stimuli compared to externally generated stimuli. The PSEs were significantly decreased in the *self-generated touch* condition compared to the *externally generated touch* condition. **e** Density plot for somatosensory attenuation (difference in the PSEs between the two conditions). **f** A lower JND value indicates a higher somatosensory precision. **g** Line plots illustrate the changes in JNDs when experiencing self-generated tactile stimuli compared to externally generated stimuli. The JNDs did not significantly differ between the *self-generated touch* and *externally generated touch* conditions. **h** Density plot for the difference in the sensory precision between the two conditions.

The psychophysical paradigm was a two-alternative forced-choice force-discrimination task that has been extensively used to assess somatosensory attenuation^15,18,24,25,70,71^. On each trial, the participants received two taps (*test* and *comparison* taps) on the pulp of their left index fingers, and they had to verbally indicate which felt stronger: the first or the second tap. The intensity of the *test* tap was set to 2 N, while the intensity of the *comparison* tap was systematically varied among seven force levels (1, 1.5, 1.75, 2, 2.25, 2.5 or 3 N). In the *externally generated touch* condition (Fig. 1a), the participants kept both of their hands relaxed while receiving the *test* tap and *comparison* taps on their left index finger. In the *self-generated touch* condition (Fig. 1b), the participants actively tapped a force sensor with their right index finger and triggered the *test* tap on their left index finger. Then, they remained relaxed while receiving the *comparison* tap. Each condition consisted of 70 trials, resulting in 140 trials per participant. The order of the intensities was randomized across participants. The order of the conditions was counterbalanced across participants. For detailed description, see Supplementary Material.

### Psychophysical fits

In each condition, the participants’ responses were fitted with a generalized linear model using a *logit* link function (Eq. 1)

1 $$p = \frac{{e^{\beta 0 + \beta 1x}}}{{1 + e^{\beta 0 + \beta 1x}}}$$

Two parameters of interest were extracted. The point of subjective equality $\left( {\rm{PSE} = - \frac{{\beta 0}}{{\beta 1}}} \right)$ represents the intensity at which the *test* tap felt as strong as the *comparison* tap (*p* = 0.5) and quantifies the participants’ perceived intensity of the *test* tap. Subsequently, somatosensory attenuation is calculated as the difference between the PSEs of the two conditions (PSE_external_ – PSE_self_)^15,18,24,25,70,71^. The just noticeable difference parameter $\left( {\rm{JND} = \frac{{{{{\mathrm{log}}}}(3)}}{{\beta 1}}} \right)$ reflects the participants’ sensitivity in the psychophysics task and quantifies their somatosensory precision in each condition. The PSE and JND are independent qualities of sensory judgments.

### Schizotypal traits

After the psychophysical task, participants completed the Schizotypal Personality Questionnaire (SPQ)^44^, a 74-item self-report schizotypy assessment instrument with excellent internal reliability (Cronbach’s alpha = 0.91) and test-retest reliability (0.82)^44^. It was developed on the basis of the nine features of schizotypal personality disorder, as defined by the DSM-III-R criteria (American Psychiatric Association, 1987)^44^. We used the three-factor model to partition the dimensions of the construct of schizotypy^60,61,63,72–75^, and we calculated the total score for the cognitive-perceptual, interpersonal and disorganized factors that reflect the positive, negative, and disorganized dimensions of schizotypy, respectively. There has been discussion as to whether schizotypy constitutes a continuous or a categorical construct^46,68,76–78^. In line with the predominant conceptualization of schizotypy as a continuous variable within the general population^46,47,50,79^, our main analysis comprised treating positive schizotypal traits as a continuous variable across the entire sample. Nonetheless, to attain methodological rigor and to account for both notions, we performed a secondary analysis treating schizotypy as a categorical variable.

### Statistical analysis

Data were analyzed using R^80^ and JASP^81^. Data normality was assessed using the Shapiro–Wilk test, and planned comparisons were made using parametric (independent or paired *t*-test) or nonparametric (Mann–Whitney or Wilcoxon) statistical tests. For each test, 95% confidence intervals (CI^95^) are reported. Depending on the data normality, effect sizes are given by Cohen’s *d* or by the matched rank biserial correlation rrb. For the ANOVAs, effect sizes are given by the partial eta-squared (*η*_*p*_^2^). Spearman correlation coefficients were used as the data were not normally distributed. Model comparison was performed using the Akaike information criterion. A Bayesian factor analysis was carried out for all statistical comparisons of our categorical analyses (default Cauchy priors with a scale of 0.707) and correlations (Kendall’s tau-b) to provide information about the level of support for the null hypothesis compared to the alternative hypothesis (BF_01_) given the data. All statistical tests were two-tailed.

## Results

### Somatosensory attenuation and precision across the entire sample

The PSE was significantly lower in the *self-generated touch* condition than in the *externally generated touch* condition across the entire sample: *n* = 100, *V* = 625, *p* < 0.001, CI^95^ = [−0.185, −0.105], rrb = −0.747, BF_01_ < 0.001 (Fig. 1c, d). This indicates that self-generated tactile stimuli felt weaker than externally generated stimuli of identical intensity, replicating previous findings^15,18,24,25,70,71^. When removing the extreme PSE value of one participant (Fig. 1c), the same results were obtained: *n* = 99, *V* = 625, *p* < 0.001, CI^95^ = [−0.180, −0.105], rrb = −0.742, BF_01_ < 0.001. Attenuation was observed in 80% of participants (Fig. 1e).

JNDs did not significantly differ between the two conditions: *n* = 100, *V* = 2592, *p* = 0.335, CI^95^ = [−0.01, 0.03], rrb = 0.113 (Fig. 1f, g). This was strongly supported by a Bayesian analysis (BF_01_ = 5.417) indicating that self-generated and externally generated taps were perceived with similar sensory precision, in line with previous studies^24,25^. When removing the extreme JND value of one participant (Fig. 1f), the same results were obtained: *n* = 99, *V* = 2592, *p* = 0.247, CI^95^ = [−0.005, 0.03], rrb = 0.137, BF_01_ = 3.480. As seen in Fig. 1h, approximately half of the participants increased and half decreased their JNDs between the conditions (44% increased, 52% decreased, 4% remained unchanged).

No significant correlation was observed between the PSEs and JNDs in either the *self-generated touch* condition (*n* = 100, rho = 0.079, *p* = 0.437) or in the *externally generated touch* condition (*n* = 100, rho = 0.046, *p* = 0.647), and this was strongly confirmed by a Bayesian analysis (BF_01_ = 5.452 for the *self-generated touch* condition, and BF_01_ = 6.560 for the *externally generated touch* condition). This corroborates the notion that sensory magnitude (PSE) and precision (JND) are independent measures, and is in line with previous findings^25^. No order effects were detected neither in the PSEs nor in the JNDs. Supplementary Material shows all individual fits.

### Schizotypal traits and somatosensory attenuation

**Fig. 2:**
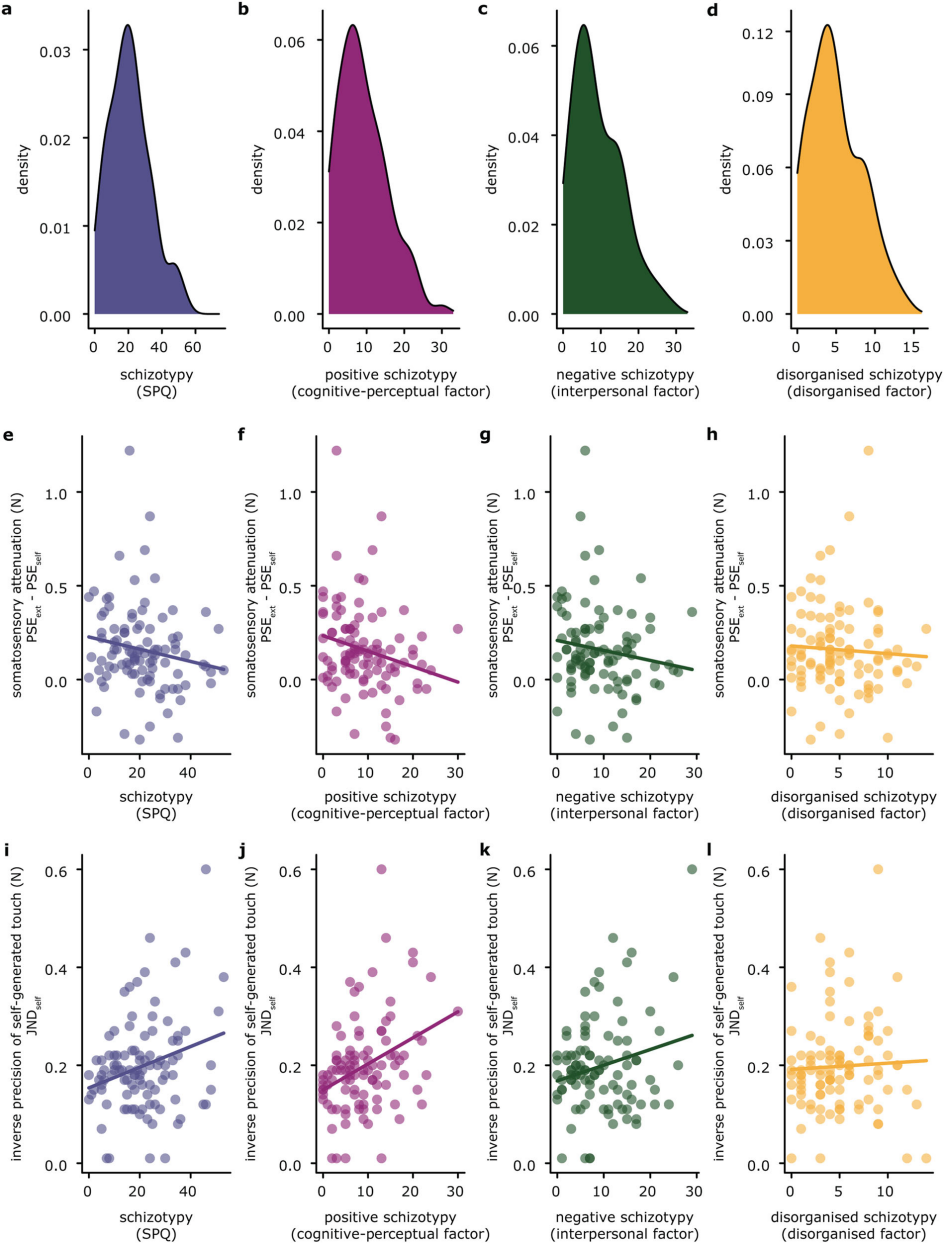
Schizotypal traits and somatosensory attenuation and precision. **a**–**d** Density plots of the Schizotypal Personality Questionnaire (SPQ) scores (possible score ranges: total, 0–74; cognitive-perceptual, 0–33; interpersonal, 0–33; disorganized, 0–16). **e**–**h** Correlations between the Schizotypal Personality Questionnaire (SPQ) scores and somatosensory attenuation. **i**–**l** Correlations between the Schizotypal Personality Questionnaire (SPQ) scores and the inverse somatosensory precision of self-generated touch (JND). Note that the *y*-axis displays the JNDs (i.e., the inverse somatosensory precision). **e**–**l** Regression lines are shown for illustrative purposes only, since we used the Spearman correlation coefficient to calculate the correlation between the variables. The positive schizotypy was the only dimension that significantly correlated with somatosensory attenuation and the precision of self-generated touch.

Figure 2a–d shows the distribution of the total SPQ scores (*μ* = 20.87, *σ* = 12.165, range = 0–53, Cronbach’s alpha = 0.821), as well as those of the cognitive-perceptual, interpersonal, and disorganized factors in our sample. Our schizotypy distributions were very similar to those of previous studies using random sampling methods, both in terms of mean and variability (e.g.,^68,82^). The sample had comparable levels of positive, negative, and disorganized schizotypy. For details, see Supplementary Material.

Confirming our first hypothesis, we observed a negative correlation between somatosensory attenuation and schizotypal traits (*n* = 100, rho = −0.215, *p* = 0.031, BF_01_ = 0.865) (Fig. 2e), which was driven by the scores of the cognitive-perceptual factor (i.e., the positive dimension of schizotypy) (Fig. 2f): *n* = 100, rho = −0.259, *p* = 0.009, BF_01_ = 0.243. This means that the higher the positive schizotypal traits of the participants, the lower their somatosensory attenuation. The individual PSEs did not significantly correlate with positive schizotypy (*self-generated touch* condition: *n* = 100, rho = −0.097, *p* = 0.335; *externally generated touch* condition: *n* = 100, rho = −0.180, *p* = 0.074). The absence of these significant correlations was supported by a Bayesian analysis (BF_01_ = 4.794 for the *self-generated touch* condition and BF_01_ = 1.502 for the *externally generated touch* condition), indicating that positive schizotypal traits are associated with the perceived *difference* between the intensities of a self-generated and an externally generated touch (i.e., somatosensory attenuation). Critically, the relationship between attenuation and schizotypy was found only for positive schizotypy and not for the negative (i.e., interpersonal factor) (*n* = 100, rho = −0.179, *p* = 0.074) (Fig. 2g) or the disorganized dimension (i.e., disorganized factor) (*n* = 100, rho = −0.106, *p* = 0.294) (Fig. 2h), and a Bayesian analysis further supported the absence of these relationships (BF_01_ = 1.552 for the negative and BF_01_ = 4.337 for the disorganized dimension).

To test the predictive power of each schizotypy dimension on somatosensory attenuation, we built three different linear models with the positive, negative, and disorganized schizotypy as independent predictors of somatosensory attenuation (difference in the PSEs), respectively. In all three models, three participants (out of 100) were considered outlier values based on a normal Q-Q plot and were removed. Residual errors were normally distributed. When comparing the three models, the Akaike information criterion favored the one with positive schizotypy (*AIC* = −55.548), followed by the one with disorganized schizotypy (*AIC* = −49.981) and then the one with negative schizotypy (*AIC* = −49.961). To further test whether positive schizotypy was a better predictor of somatosensory attenuation, over-and-above the other two schizotypy dimensions, we built a model with all three schizotypal dimensions included as simultaneous predictors. All three regressors had low variance inflation factors (<1.86) and the residual errors were normally distributed. Positive schizotypy was a significant negative regressor on somatosensory attenuation (*n* = 97, *t* = −2.292, *p* = 0.024) but neither the negative (*n* = 97, *t* = 0.547, *p* = 0.586), nor the disorganized dimensions of schizotypy (*n* = 97, *t* = −0.372, *p* = 0.711) were significant predictors of attenuation. These results suggest that the predictive power of positive schizotypy is higher than that of negative and disorganized schizotypy in accounting for the attenuation effect and demonstrates the specificity of the positive subscale.

### Schizotypal traits and somatosensory precision

Confirming our second hypothesis, we observed a positive correlation between the JND of self-generated touch and positive schizotypal traits (*n* = 100, rho = 0.339, *p* < 0.001, BF_01_ = 0.018) (Fig. 2j), which effectively is a negative correlation between the somatosensory precision of self-generated touch and positive schizotypal traits. In other words, the higher the positive schizotypal traits of the participants, the lower their somatosensory precision of self-generated touch. In contrast, somatosensory precision for externally generated touch did not correlate with positive schizotypy (*n* = 100, rho = 0.114, *p* = 0.257, BF_01_ = 3.639), suggesting that positive schizotypy does not generically influence the precision with which touch is perceived but only that of self-generated touch. Finally, the somatosensory precision of self-generated touch significantly correlated only with positive schizotypy but not with the full SPQ (*n* = 100, rho = 0.167, *p* = 0.096) (Fig. 2i), or the negative (*n* = 100, rho = 0.039, *p* = 0.699) (Fig. 2k) or disorganized dimension (*n* = 100, rho = 0.092, *p* = 0.364) (Fig. 2l). As above, the Bayesian analyses strongly supported the absence of these relationships (BF_01_ = 6.600 for the negative dimension and BF_01_ = 4.682 for the disorganized dimension).

As with somatosensory attenuation, to test the predictive power of each schizotypy dimension on the precision of self-generated touch, we built three different linear models with the positive, negative, and disorganized schizotypy as independent predictors of somatosensory precision (JND_self_), respectively. In all three models, three participants (out of 100) were considered outlier values based on a normal Q-Q plot and were removed. Residual errors were normally distributed for the positive and the negative schizotypy. They were not normally distributed for the disorganized dimension but followed a bell-shaped curve. When comparing the three models, the Akaike information criterion favored the one with positive schizotypy (*AIC* = −218.68), followed by the one with negative schizotypy (*AIC* = −207.732) and then the one with disorganized schizotypy (*AIC* = −207.322). To further test whether positive schizotypy was a better predictor of the precision of self-generated touch, over-and-above the other two schizotypy dimensions, we built a model with all three schizotypal dimensions included as simultaneous predictors. All three regressors had low variance inflation factors (<1.69). The residuals were not normally distributed but followed a bell-shaped curve. Similar to somatosensory attenuation, positive schizotypy was a significant regressor on somatosensory precision (*n* = 97, *t* = 8.436, *p* = 0.005) while neither the negative (*n* = 97, *t* = 0.353, *p* = 0.725), nor the disorganized dimensions of schizotypy (*n* = 97, *t* = −1.062, *p* = 0.291) were significant regressors. Together, these results suggest that the predictive power of positive schizotypy is higher than that of negative and disorganized schizotypy in accounting for the lower precision of self-generated touch and demonstrates the specificity of the positive subscale.

### Schizotypy as a categorical variable

**Fig. 3:**
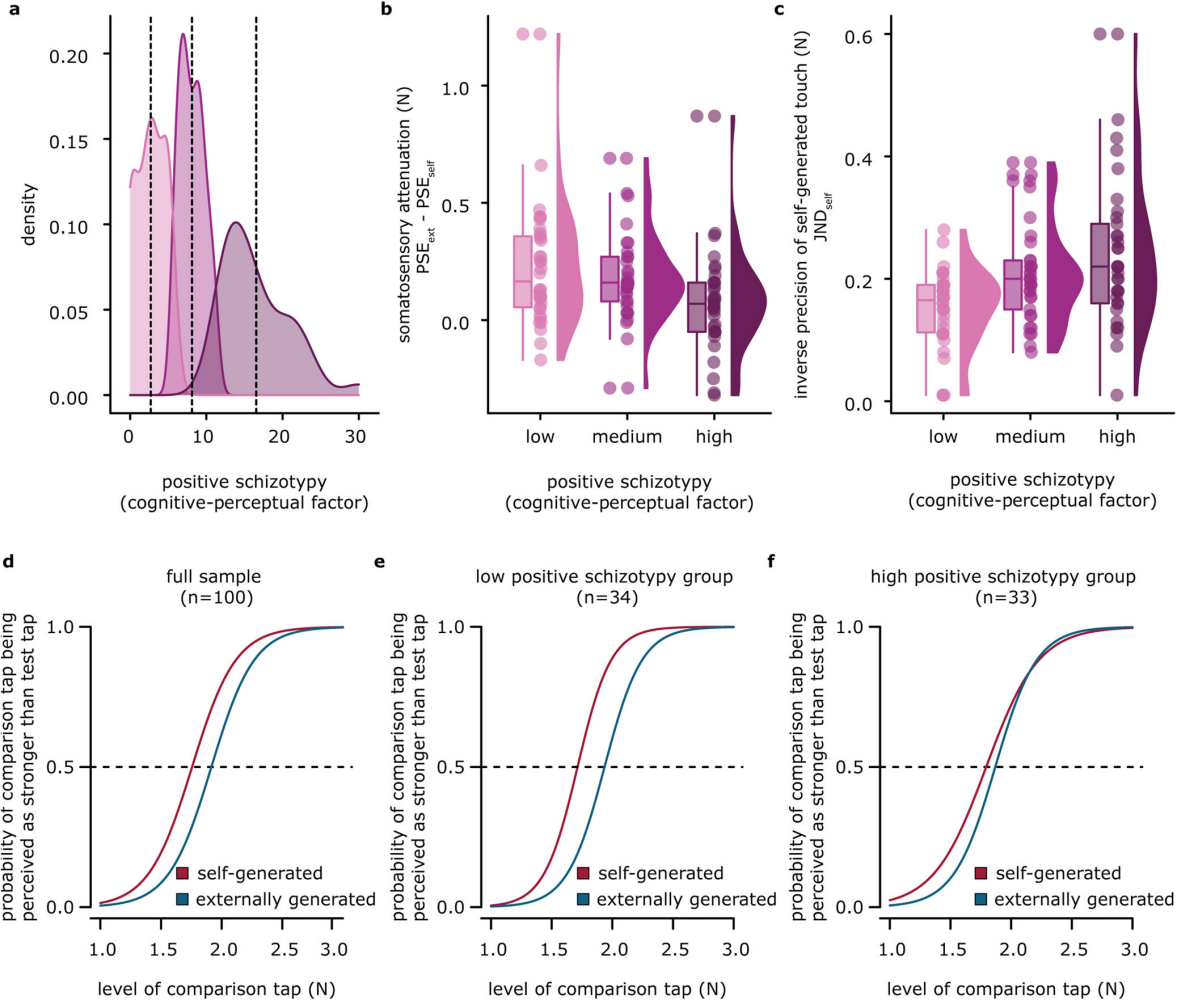
Somatosensory attenuation and precision in individuals with low, medium, and high positive schizotypal traits. **a** Density plots for the three positive schizotypy subgroups of our sample. Vertical dotted lines indicate the mean of each subgroup. **b** The high positive schizotypy group showed significantly less somatosensory attenuation than the low positive schizotypy group. **c** The high positive schizotypy group showed significantly less somatosensory precision (significantly higher JND) in the *self-generated touch* condition than the low schizotypy group. **d** Group psychometric fits using the total sample. The fits for each condition were generated using the mean PSE and the mean JND across participants. **e**, **f** Group psychometric fits for the low (**e**) and the high (**f**) positive schizotypy groups. The high positive schizotypy group shows a substantially smaller shift in the curves between the *self-generated* and *externally generated* touch conditions (i.e., less attenuation) and a flatter slope for the *self-generated touch* condition (i.e., higher JND).

Finally, we treated positive schizotypal traits as a categorical variable. Given the absence of established cut-off values for the SPQ estimates, we split the sample into 3 subgroups with equal numbers of participants based on their scores in the cognitive-perceptual factor: the low (*n*_low_ = 34), medium (*n*_med_ = 33), and high (*n*_high_ = 33) positive schizotypy groups (Fig. 3a). This approach was deemed appropriate to discern the differences between the two extremes (i.e., low and high).

For the PSEs, a mixed ANOVA with condition (*self-generated* versus *externally generated*) as the within-subjects’ factor, and positive schizotypy group (*high* versus *low*) as the between subjects’ factor revealed a significant main effect of condition (*F*(1,65) = 25.94, *p* < 0.001, *η*_*p*_^2^ = 0.285), a non-significant effect of schizotypy group (*F*(1,65) = 0.041, *p* = 0.840, *η*_*p*_^*2*^ < 0.001), and a significant interaction (*F*(1,65) = 6.402, *p* = 0.014, *η*_*p*_^2^ = 0.090). The interaction was driven by a significantly higher somatosensory attenuation for the low positive schizotypy group compared to the high positive schizotypy group (Fig. 3b): *n*_low_ = 34*, n*_high_ = 33*, W* = 770, *p* = 0.009, CI^95^ = [0.030, 0.230], rrb = 0.373, BF_01_ = 0.280.

For the JNDs, the mixed ANOVA revealed a non-significant main effect of condition (*F*(1,65) = 1.890, *p* = 0.174, *η*_*p*_^2^ = 0.028), a significant effect of schizotypy group (*F*(1,65) = 9.508, *p* = 0.003, *η*_*p*_^2^ = 0.128), and a significant interaction (*F*(1,65 = 8.346, *p* = 0.005, *η*_*p*_^2^ = 0.114). The interaction term was driven by lower JNDs in the *self-generated touch* condition for the low positive schizotypy group compared to the high positive schizotypy group (*n*_low_ = 34*, n*_high_ = 33, *t*(48.7) = −3.626, *p* < 0.001, CI^95^ = [−0.133, −0.038], Cohen’s *d* = −0.89, BF_01_ = 0.018) (Fig. 3c). In contrast, JNDs in the *externally generated touch* condition did not significantly differ between the two groups (*n*_low_ = 34*, n*_high_ = 33, *W* = 477.5, *p* = 0.296, CI^95^ = [−0.050, 0.020], rrb = −0.149, BF_01_ = 2.46).

Figure 3d–f illustrates these effects for the entire sample (Fig. 3d), the low (Fig. 3e) and the high positive schizotypy subgroups (Fig. 3f). In the entire sample, the psychometric curve shifted to the left for the *self-generated touch* condition compared to the *externally generated touch* condition without any changes in the slope; thus, self-generated touch felt weaker than external touch, but they were perceived with similar precision (Fig. 3d). Critically, as seen in Fig. 3e and f, the high positive schizotypy group showed less of a shift between the PSEs in the *self-generated* and *externally generated touch* conditions (less attenuation) and a flatter curve in the *self-generated touch* condition (higher JND) compared to the low schizotypy group.

## Discussion

The present study has two main findings. First, individuals with higher positive schizotypal traits exhibited less attenuation of their self-generated touch than individuals with low positive schizotypal traits. This result strongly mirrors previous clinical findings of reduced somatosensory attenuation in patients with schizophrenia^30–32^. This is also in line with earlier observations that nonclinical individuals with high schizotypy subjectively rate self-generated touch as more ticklish^78^ and intense^68^ than those with low schizotypy. Second, our experimental task (i.e., the force-discrimination task) enabled the measurement not only of the perceived magnitude but also of somatosensory precision and consequently, the assessment of its relationship with schizotypy. Individuals with higher positive schizotypal traits perceived self-generated touch with less sensory precision than individuals with lower positive schizotypal traits, without any effect on the precision of externally generated touch. This result indicates for the first time that high positive schizotypal traits are not accompanied by generic deficits in processing afferent somatosensory information but only self-generated somatosensory feedback and enforces the view that self-disorders lie at the core of the schizophrenia spectrum^38,39,41,83,84^. Critically, both in terms of attenuation and precision of self-generated touch, it was the positive dimension of schizotypy that drove the effects and not the negative or disorganized dimensions. This parallels the association previously observed between somatosensory attenuation and the severity of hallucinations^31^, as well as the delusional ideation^85,86^ and passivity experiences^78^ of nonclinical individuals.

Deficits in somatosensory attenuation and precision can fall within the scope of subtle neurological aberrations in sensorimotor performance^87,88^, that are present with variable severity across the psychosis continuum^89–93^. Neurological soft signs have been repeatedly associated with the negative symptoms of schizophrenia and negative schizotypy in non-clinical individuals^94–104^, and less robustly with the positive and disorganized dimensions^89,105–107^. Instead, our data revealed a relationship of somatosensory attenuation and precision only with positive schizotypy, and not with the negative and the disorganized dimensions. Consequently, our findings suggest that somatosensory attenuation and precision constitute a special category of neurological soft signs that is specifically related to the self and the positive dimension of psychotic and psychotic-like symptoms.

Our results provide important insights for understanding the mechanism underlying the positive symptoms of schizophrenia. From a computational perspective, our effects can be explained by a deficit in the internal forward model that predicts the somatosensory consequences of the movement. Earlier studies have shown that somatosensory attenuation relies on spatiotemporal motor predictions^18,19,71^ and not on postdictive processes^25,70^, and it requires conditions where the received touch can be predicted by the motor command^15,17,18,20–22,24,70^. In our study, reduced attenuation indicates that with the same motor command, the brain of an individual with high positive schizotypy does not accurately predict the sensory consequences of the voluntary movement, leading to less attenuation of the self-generated somatosensory feedback compared to an individual with low positive schizotypy. The combination of this inaccurately predicted somatosensory information with the actual somatosensory feedback further leads to the decreased precision of self-generated touch. Within a Bayesian framework where prediction corresponds to prior expectations and sensory feedback to sensory evidence^35^, our study indicates that high positive schizotypy is related to atypical prior expectations (generated by the internal forward model) and atypical combinations of prior knowledge with sensory evidence.

The cerebellum has been repeatedly implicated in predicting the sensory consequences of one’s own actions^11,70,108–112^, and we previously showed that participants with stronger functional connectivity between the cerebellum and the primary and secondary somatosensory cortices during self-generated touch compared to externally generated touch, show greater somatosensory attenuation^23^. Schizophrenia is also strongly associated with alterations in structural and functional cerebellar connectivity^113,114^. Patients show impairments in cerebellar-mediated motor tasks^115^, deficits in the integrity of the cerebellar white matter tracts^116,117^, and altered cerebellar connectivity^118–124^ and activation^125^ compared to healthy controls. Individuals at ultra-high-risk for psychosis have decreased resting-state cerebellocortical connectivity compared to controls^126^, and their functional and structural cerebellocortical connectivity relates to their positive symptom progression^127^. Based on our findings, we speculate that positive schizotypy and consequently the positive symptoms of schizophrenia are related to altered corticocerebellar connectivity.

Future efforts should exploit the perception of self-generated somatosensation as a potential indicator of psychosis proneness. In contrast to other markers, including prepulse inhibition, mismatch negativity and the P300^128^, which reflect deficits in processing externally generated information in schizophrenia, our results emphasize deficits in processing self-generated information. Furthermore, given that the positive symptoms in the prodromal phase are highly predictive of the transition from a high-risk state to schizophrenia^129,130^, self-generated somatosensation could function as a sign of neurocognitive impairment that, when combined with other genetic, biochemical and neuroimaging markers^131,132^, forms a multilayered ‘signature’ for schizophrenia liability. This could enable early detection of psychosis proneness using objective measures that are not susceptible to self-report bias like scale-based measures. So far, this perspective is still at a premature stage and the implementation in clinical settings is far from complete. Undoubtedly, appropriate clinical contextualization and validation through future longitudinal studies are necessary. Nonetheless, the present study suggests that deficits in processing self-generated touch can indicate increased liability for schizophrenia.

## Supplementary information

Supplementary materialś
