# Supplementary material for: The positive dimension of schizotypy is associated with a reduced attenuation and precision of self-generated touch
Source: Schizophrenia (Heidelb). 2022 Jun 29;8(1):57. doi: 10.1038/s41537-022-00264-6 (PMC9261081; doi:10.1038/s41537-022-00264-6)
Supplement: Supplementary file 1 — Supplementary materialś [file 41537_2022_264_MOESM1_ESM.docx]

**Supplementary Material**

***Experimental procedures***

Participants rested their left hand, palm up, with the index finger placed inside a molded support (**Figure 1**). On each trial, a DC electric motor (Maxon EC Motor EC 90 flat; manufactured in Switzerland) delivered two brief (100 ms) forces on the pulp of participants’ left index finger through a cylindrical probe (25 mm height) with a flat aluminum surface (20 mm diameter) attached to the motor’s lever. We refer to the first force as the *test* tap and to the second force as the *comparison* tap. The intensity of the *test* tap was set to 2 N, while the intensity of the *comparison* tap was systematically varied among seven force levels (1, 1.5, 1.75, 2, 2.25, 2.5 or 3 N). On each trial, participants verbally reported which tap felt stronger: the *test* tap or the *comparison* tap. A force sensor (FSG15N1A, Honeywell Inc.; diameter, 5 mm; minimum resolution, 0.01 N; response time, 1 ms; measurement range, 0–15 N) was placed within the probe to record the forces exerted on the left index finger. A force of 0.1 N was constantly applied to the participants’ left index finger to ensure accurate force intensities.

There were two experimental conditions, the order of which was counterbalanced across participants. In the *externally generated touch* condition, participants relaxed their right arm and passively received the two taps on their left index finger (**Figure 1a**). The *test* tap was delivered 800 ms after an auditory ‘go’ cue, and the *comparison* tap was delivered after a random delay (800 ms – 1500 ms) from the end of the *test* tap. In the *self-generated touch* condition, participants actively tapped with their right index finger a force sensor placed above (but not in contact with) the probe after the auditory ‘go’ cue (**Figure 1b**). They were instructed to tap the sensor “approximately as strong as when they tapped the surface of their smartphone”. The tap of their right index finger triggered the *test* tap on their left index finger with an intrinsic delay of 36 ms.

Each condition consisted of 70 trials, resulting in 140 trials per participant. The order of the intensities was randomized across participants. In both conditions, the view of the pulp of the left index finger was occluded, and participants fixated on a cross 2 meters in front of them. Any sounds created by the motor or the participants’ taps were suppressed by administering white noise through a pair of headphones. Before the experiment, the participants were instructed to avoid balancing their responses. If the intensity of the two taps felt very similar, they were explicitly told that they had to guess. No feedback was provided about their performance.

Previous studies investigating somatosensory attenuation have compared the *self-generated touch* condition with an *externally generated touch* condition or a self-generated ‘delayed’ condition that includes a delay between the tap of the right index finger and the *test* tap on the left index finger ^1–6^. Here, we chose to include the externally generated touch condition to gain information not only about how schizotypal traits relate to self-generated touch, but also externally generated touch.

***Data Processing***

Before fitting the responses, the values of the applied *comparison* taps were binned to the closest value with respect to their theoretical values (1, 1.5, 1.75, 2, 2.25, 2.5 or 3 N). After data collection, 951 trials out of 14000 (6.8%) were rejected, either because the intensity of the *test* tap (2 N) was not applied accurately (*test* tap < 1.85 N or *test* tap > 2.15 N) or due to missing responses.

***Individual Fits***


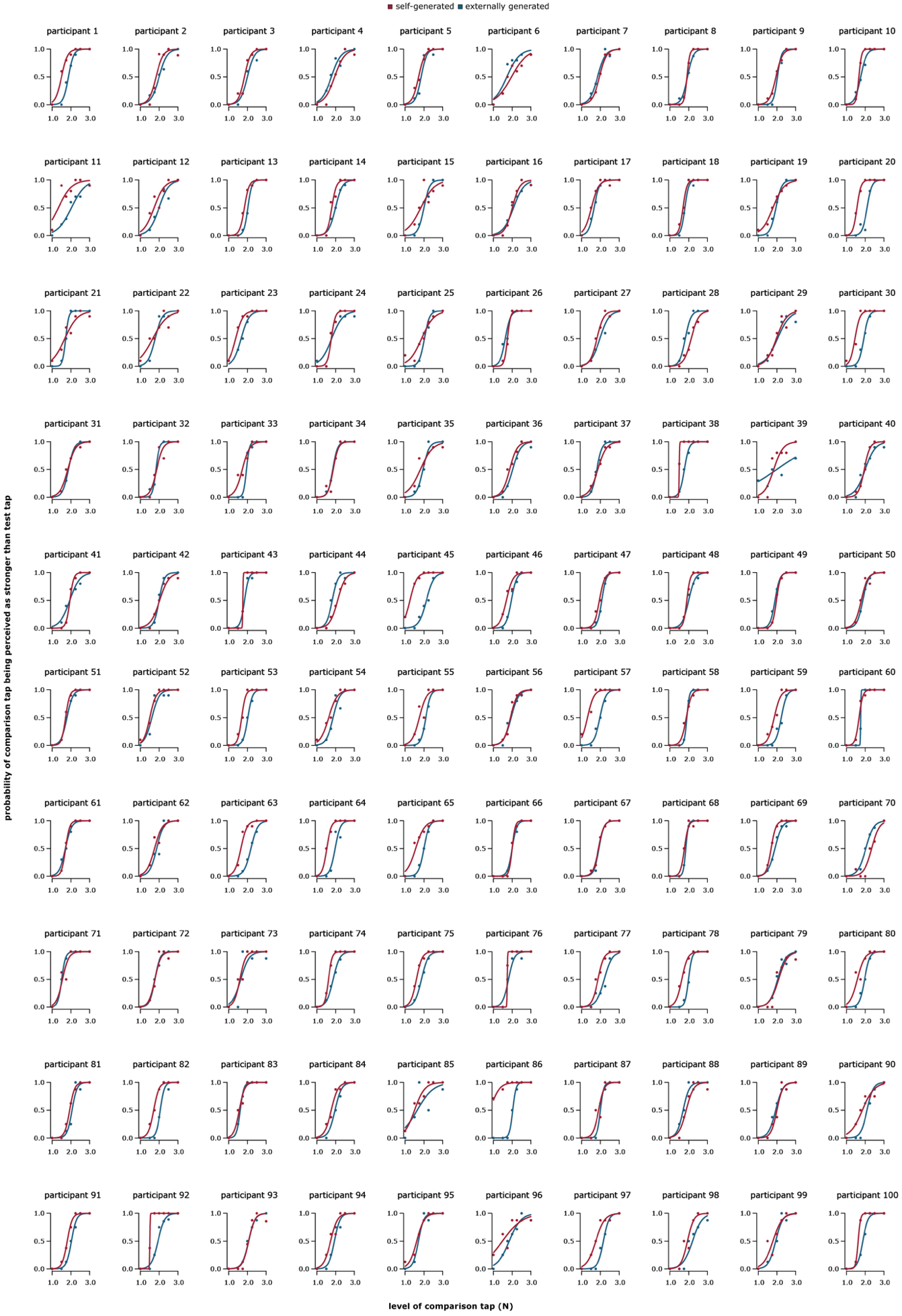
 **Figure S1. Fitted logistic models based on the participants’ responses under each condition.**

***Schizotypal Personality Traits***

**Table S1** shows the full descriptive statistics for the SPQ and **Figure S2** illustrates the correlations between its subscales.

**Table S1. Descriptive statistics for the SPQ, its factors and its subscales.**

| **SPQ Parameter** | **Mean** | **Standard Deviation** | **Variance** | **Range** |
| --- | --- | --- | --- | --- |
| *SPQ total* | 20.870 | 12.165 | 147.993 | 0-53 |
| **Factors** | | | | |
| *Cognitive-Perceptual* | 9.060 | 6.412 | 41.107 | 0-30 |
| *Interpersonal* | 9.280 | 6.566 | 43.113 | 0-29 |
| *Disorganized* | 4.940 | 3.381 | 11.431 | 0-14 |
| **Subscales** | | | | |
| *Ideas of Reference* | 3.130 | 2.489 | 6.195 | 0-9 |
| *Odd Beliefs or Magical Thinking* | 1.450 | 1.696 | 2.876 | 0-7 |
| *Unusual Perceptual Experiences* | 2.070 | 1.908 | 3.642 | 0-8 |
| *Odd or*  *Eccentric Behaviour* | 1.630 | 1.868 | 3.488 | 0-6 |
| *Excessive Social Anxiety* | 2.870 | 2.460 | 6.054 | 0-8 |
| *No Close Friends* | 2.190 | 2.398 | 5.751 | 0-9 |
| *Odd Speech* | 3.310 | 2.214 | 4.903 | 0-9 |
| *Constricted Affect* | 1.810 | 1.756 | 3.085 | 0-7 |
| *Suspiciousness* | 2.410 | 2.000 | 4.002 | 0-8 |


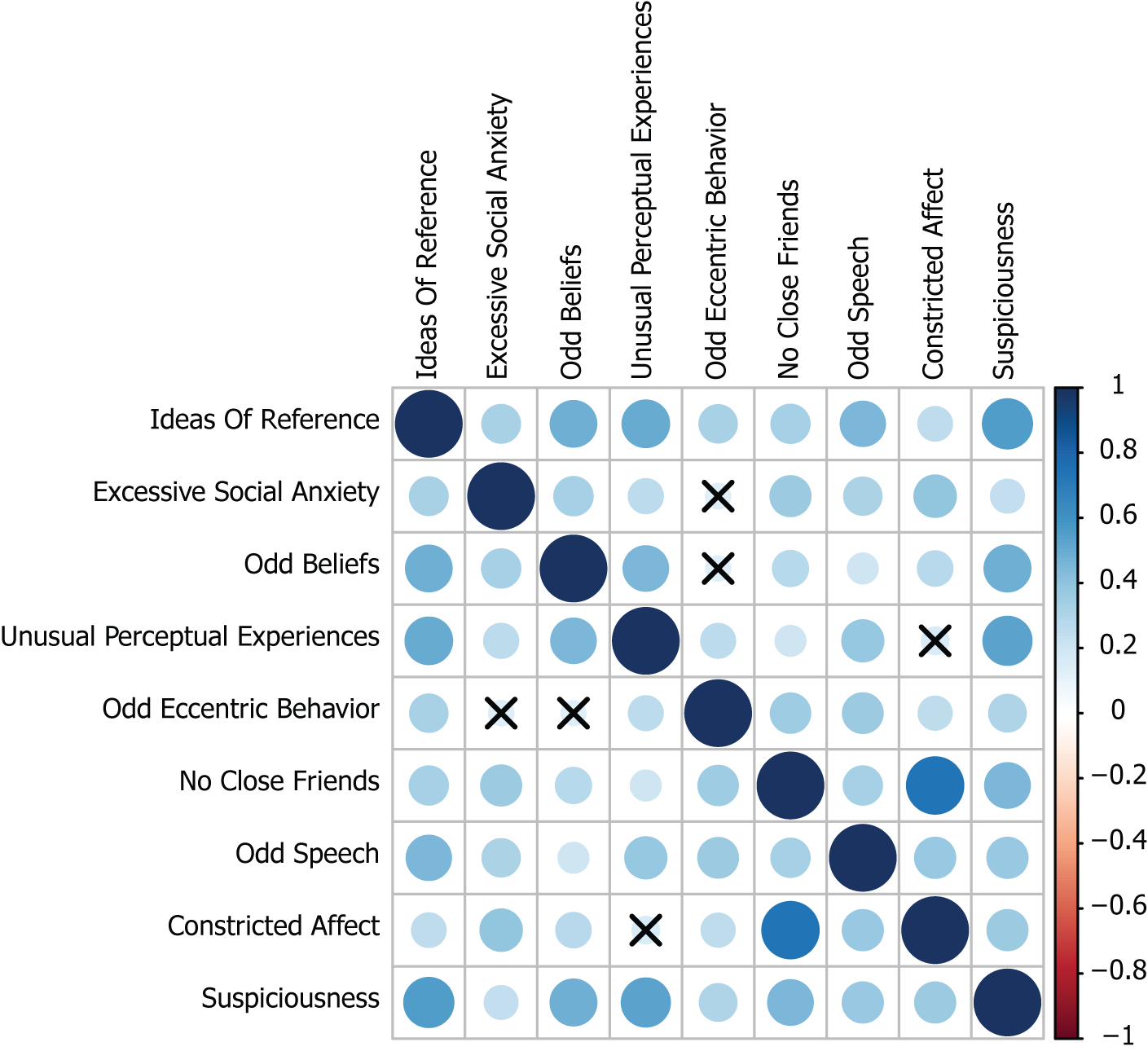


**Figure S2. Correlations between the scores of all the SPQ subscales.** The correlations have been calculated using the Spearman correlation coefficient and the *p*-values have been corrected for False Discovery Rate (FDR). X denotes a statistically non-significant correlation.

Our sample had comparable levels of positive, negative and disorganized schizotypal traits, as can be seen in **Figure 2b-c**. A *Levene*’s test for Homogeneity of Variance revealed no significant difference between the variances of the positive and the negative schizotypy (cognitive-perceptual and interpersonal, *F*(1,198) = 0.115, *p* = 0.735). To compare the variances between the disorganized schizotypy scores and those of the cognitive perceptual or the interpersonal schizotypy, we first rescaled the disorganized scores (range: 0-16) to the same range as that of cognitive perceptual and interpersonal schizotypal scores (range: 0-33). We then performed the *Levene*’s test which revealed no significant difference in the variances of the cognitive-perceptual and the disorganized factors (*F*(1,198) = 0.691, *p* = 0.407), as well as between the interpersonal and disorganized factors (*F*(1,198) = 0.242, *p* = 0.624).

***Somatosensory attenuation and precision in relation to negative and disorganized schizotypy***

To control whether the effects between somatosensory attenuation and precision are specific to the positive schizotypal traits, we repeated our categorical analysis for the negative and disorganized dimension of schizotypy.

***Negative schizotypy as a categorical variable***

We divided our sample into 3 subgroups: the low (*n_low_ = 34*), medium (*n_med_* = 33) and high (*n_high_* = 33) negative schizotypy groups (**Figure S3a**). For the PSEs, a mixed ANOVA with condition (*self-generated* versus *externally generated*) as the within-subjects’ factor, and negative schizotypy group (*high* versus *low*) as the between subjects’ factor revealed a significant main effect of condition (*F*(1,65) = 31.64, *p* < 0.001, *η_p_^2^* = 0.327), a non-significant effect of schizotypy group (*F*(1,65) = 0.012, *p* = 0.912, *η_p_^2^* < 0.001), and a non-significant interaction (*F*(1,65) = 2.190, *p* = 0.144, *η_p_^2^* = 0.033). That is, there was no significant difference between the low and high negative schizotypy in terms of somatosensory attenuation (**Figure S3b**): *n_low_ = 34, n_high_ =* 33*, t*(63.5) = 1.484, *p* = 0.143, *CI^95^* = [-0.025, 0.167], *Cohen’s d* = 0.362, *BF_01_* = 1.58. For the JNDs, the mixed ANOVA revealed a non-significant main effect of condition (*F*(1,65) = 0.021, *p =* 0.885, *η_p_^2^* < 0.001), a non-significant effect of schizotypy group (*F*(1,65) = 0.008, *p* = 0.930, *η_p_^2^* < 0.001), and a non-significant interaction (*F*(1,65) = 2.964, *p* = 0.090, *η_p_^2^* = 0.044). That is, there was no significant difference between the low and high negative schizotypy in terms of somatosensory precision: *n_low_ = 34, n_high_ =* 33*, W* = 561.5, *p* = 1, *CI^95^* = [-0.04, 0.04], *rrb* < 0.001, *BF_01_* = 3.680 (**Figure S3c**).

***Disorganized schizotypy as a categorical variable***

We divided our sample into 3 subgroups: the low (*n_low_ = 34*), medium (*n_med_* = 33) and high (*n_high_* = 33) disorganized schizotypy groups (**Figure S4a**). A mixed ANOVA with condition (*self-generated* versus *externally generated*) as the within-subjects’ factor, and disorganized schizotypy group (*high* versus *low*) as the between subjects’ factor revealed a significant main effect of condition (*F*(1,65) = 27.616, *p* < 0.001, *η_p_^2^* = 0.298), a non-significant effect of schizotypy group (*F*(1,65) = 0.325, *p* = 0.571, *η_p_^2^* = 0.005, and a non-significant interaction (*F*(1,65) = 0.034, *p* = 0.854, *η_p_^2^* < 0.001). That is, there was no significant difference between the low and high disorganized schizotypy in terms of somatosensory attenuation (**Figure S4b**): *n_low_ = 34, n_high_ =* 33*, W* = 628, *p* = 0.404, *CI^95^* = [-0.050, 0.130], *rrb* = 0.119, *BF_01_* = 3.368. For the JNDs, the mixed ANOVA revealed a non-significant main effect of condition (*F*(1,65) = 0.014, *p* = 0.906, *η_p_^2^* < 0.001), a non-significant effect of schizotypy group (*F*(1,65) = 0.147, *p* = 0.703, *η_p_^2^* = 0.002), and a non-significant interaction (*F*(1,65) = 1.500, *p* = 0.225, *η_p_^2^* = 0.023). That is, there was no significant difference between the low and high disorganized schizotypy in terms of somatosensory precision: *n_low_ = 34, n_high_ =* 33*, W* = 460.5, *p* = 0.209, *CI^95^* = [-0.070, 0.010], *rrb* = -0.179, *BF_01_* = 2.389 (**Figure S4c**).

***
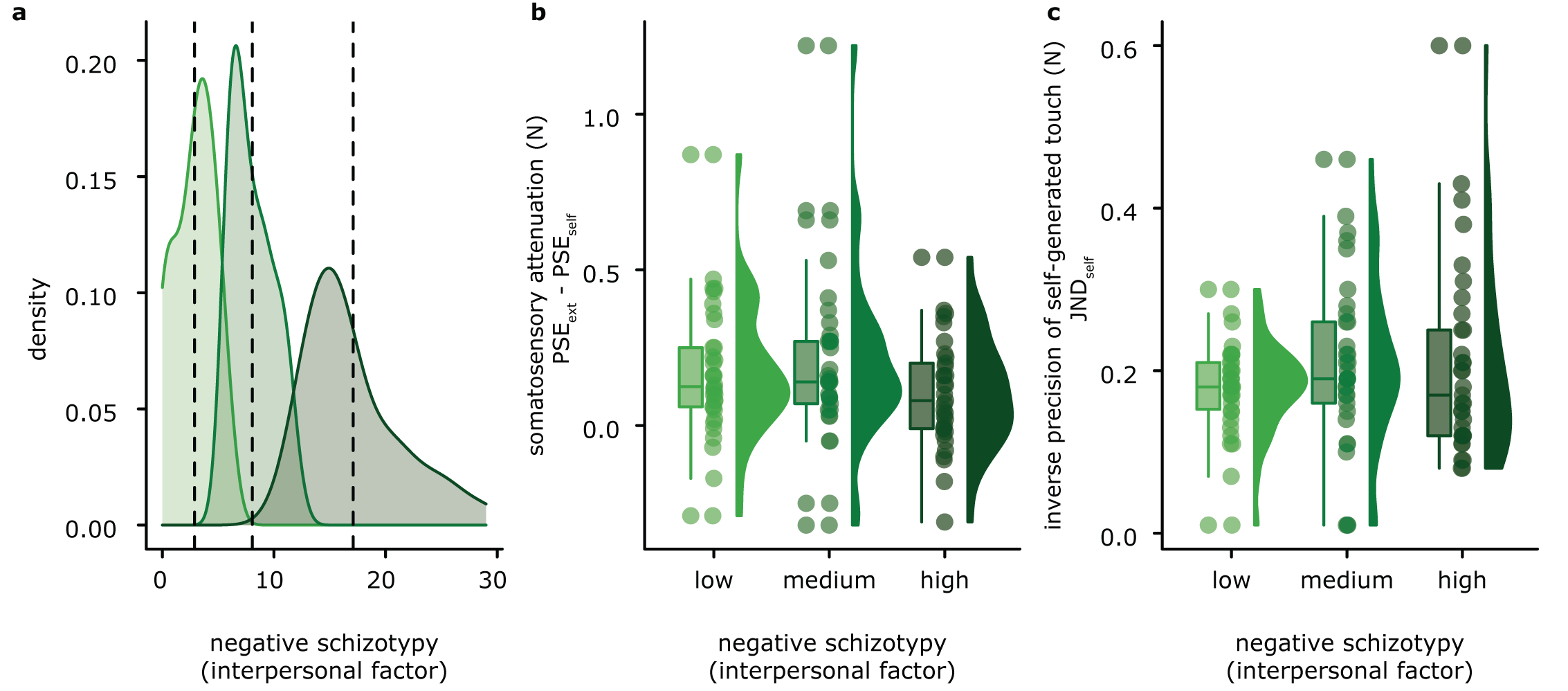
***

**Figure S3. Somatosensory attenuation and precision in individuals with low, medium, and high negative schizotypal traits. (a)** Density plots for the three schizotypy subgroups of our sample. Vertical dotted lines indicate the mean of each subgroup. **(b)** The boxplots show the median and interquartile ranges for somatosensory attenuation, the jittered points denote the raw data, and the violin plots display the full distribution of the data in each group. No significant differences in somatosensory attenuation were observed between the three groups. **(c)** The boxplots show the median and interquartile ranges for the JND in the *self-generated touch* condition, the jittered points denote the raw data, and the violin plots display the full distribution of the data in each group. No significant differences in somatosensory precision were observed between the three groups.


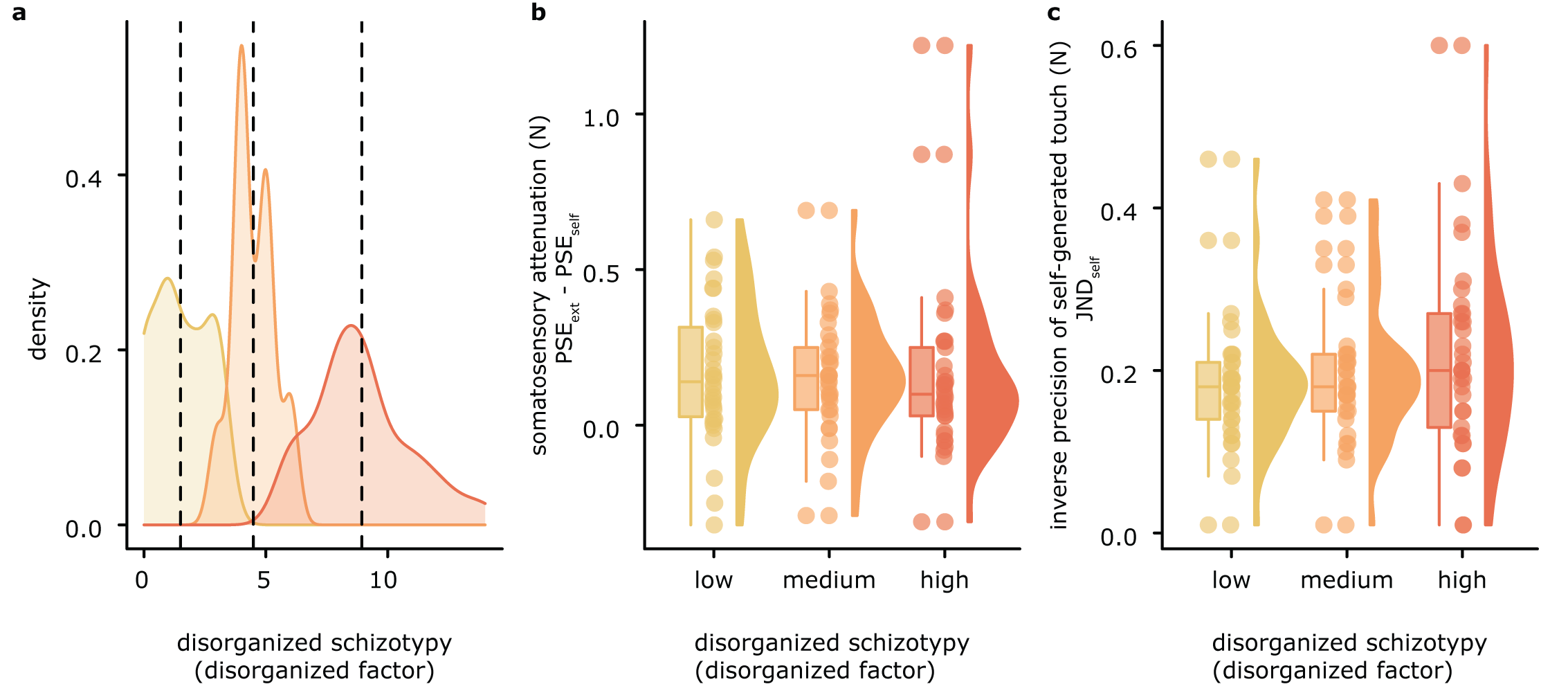


**Figure S4. Somatosensory attenuation and precision in individuals with low, medium, and high disorganized schizotypal traits. (a)** Density plots for the three schizotypy subgroups of our sample. Vertical dotted lines indicate the mean of each subgroup. **(b)** The boxplots show the median and interquartile ranges for somatosensory attenuation, the jittered points denote the raw data, and the violin plots display the full distribution of the data in each group. No significant differences in somatosensory attenuation were observed between the three groups. **(c)** The boxplots show the median and interquartile ranges for the JND in the *self-generated touch* condition, the jittered points denote the raw data, and the violin plots display the full distribution of the data in each group. No significant differences in somatosensory precision were observed between the three groups.

**References**

1. Bays, P. M., Wolpert, D. M. & Flanagan, J. R. Perception of the consequences of self-action is temporally tuned and event driven. *Curr. Biol.* **15**, 1125–1128 (2005).

2. Bays, P. M., Flanagan, J. R. & Wolpert, D. M. Attenuation of self-generated tactile sensations is predictive, not postdictive. *PLoS Biol.* **4**, 281–284 (2006).

3. Kilteni, K., Houborg, C. & Ehrsson, H. H. Rapid learning and unlearning of predicted sensory delays in self-generated touch. *Elife* **8**, 1–17 (2019).

4. Kilteni, K., Engeler, P. & Ehrsson, H. H. Efference Copy Is Necessary for the Attenuation of Self-Generated Touch. *iScience* **23**, 100843 (2020).

5. Kilteni, K. & Ehrsson, H. H. Predictive attenuation of touch and tactile gating are distinct perceptual phenomena. *iScience* **25**, 104077 (2022).

6. Kilteni, K., Engeler, P., Boberg, I., Maurex, L. & Ehrsson, H. H. No evidence for somatosensory attenuation during action observation of self-touch. *Eur. J. Neurosci.* **54**, 6422–6444 (2021).
